# Network topological determinants of pathogen spread

**DOI:** 10.1038/s41598-022-11786-5

**Published:** 2022-05-11

**Authors:** María Pérez-Ortiz, Petru Manescu, Fabio Caccioli, Delmiro Fernández-Reyes, Parashkev Nachev, John Shawe-Taylor

**Affiliations:** 1 Department of Computer Science, University College London, London, UK; 2 Institute of Neurology, University College London, London, UK

**Keywords:** Mathematics and computing, Diseases

## Abstract

How do we best constrain social interactions to decrease transmission of communicable diseases? Indiscriminate suppression is unsustainable long term and presupposes that all interactions carry equal importance. Instead, transmission within a social network has been shown to be determined by its topology. In this paper, we deploy simulations to understand and quantify the impact on disease transmission of a set of topological network features, building a dataset of 9000 interaction graphs using generators of different types of synthetic social networks. Independently of the topology of the network, we maintain constant the total volume of social interactions in our simulations, to show how even with the same social contact some network structures are more or less resilient to the spread. We find a suitable intervention to be specific suppression of unfamiliar and casual interactions that contribute to the network’s global efficiency. This is, pathogen spread is significantly reduced by limiting specific kinds of contact rather than their global number. Our numerical studies might inspire further investigation in connection to public health, as an integrative framework to craft and evaluate social interventions in communicable diseases with different social graphs or as a highlight of network metrics that should be captured in social studies.

## Introduction

The contact patterns that underlie disease transmission naturally form a network, where links join individuals that interact, and disease spreads along links. Computational models of infectious disease can support scenario analysis during epidemic outbreaks necessary to devise effective public health interventions. All epidemiological models make assumptions about the underlying network of interactions. Contact network models mathematically formalize this intuitive concept, so that epidemiological calculations explicitly consider complex patterns of interactions^1^.

Recent studies^2^ have shown that networks with equal number of nodes and edges, but different network structure (e.g. path length and clustering) lead to different infection curves. Most of the theoretical literature on this topic^3–8^ focuses on the effect on pathogen spread of individual network properties (e.g. degree distribution, assortative mixing or clustering), typically analyzed by controlled numerical experiments or analytical calculations, where only the property of interest is varied. However, in a realistic context, altering the structure of a network means simultaneously changing different network characteristics. This is because when one perturbs a network, increasing the variance of the degree distribution, for example, many other network metrics can also change, entangling the effect of different network metrics on spread. Even more, the size and complexity of the space of possible network characteristics makes the derivation of optimal metrics of spread from empirical data infeasible, for any candidate model is bound to be under determined by the scale and fidelity of available data. Rather we need large-scale simulations spanning the horizon of network parameters within which a network topology is bound to lie. Our simulations of the spread of a pathogen using a contact network model on more than 9000 synthetic social networks are a first step in this direction. Although all of the networks considered are synthetic, the families from which they are sampled have been shown to be representative in some instances of human interaction networks. Importantly, the number of social interactions per time step is kept constant for all of our simulations, independently of the network topology and average degree. That is, the same number of social interactions occur on average at each time step of the simulations (i.e., equal to the number of individuals). Note, however, that there are differences across individuals (we assume a linear relationship between the individual average degree and their number of interactions per time step)^9^. Thus, the differences reported across our simulations stem from which individuals in the population interact, rather than the total number of interactions.

The analysis of our large-scale simulations demonstrate that metrics that were found to be predictive of the spread in the literature (through experiments that only varied the metric of interest), are not predictive when one considers a larger variety of network topologies. Instead, we show that specific suppression of unfamiliar and casual interactions that contribute to the network’s global efficiency is a well suited intervention. Our conclusions not only validate previous findings on network theory and epidemiology^2,4,10,11^, but they also provide a valuable and integrative view of the impact of different network factors on communicable disease spread. The results from our simulations, which include statistics of network metrics and pathogen spread, together with parallel processing python code to run these simulations, are available at our project repository (https://github.com/mperezortiz/topology_spread). The code can be easily extended to other network families, network metrics and contact network models. Note that our simulator makes many simplifying assumptions and many other factors that are not considered in our experiments could influence the spread in real-world settings. However, this paper is mainly concerned with studying network topological determinants of pathogen spread in a large-scale experiment covering a wide range of networks, rather than a specific real-world network. Our aim is to motivate the need for such large-scale experiments when considering determinants of pathogen spread. However, it is still important to emphasize that further experiments are needed in this direction to find determinants that may apply to a wide range of cases and connect to the real-world.

## Results

### Method overview

We generate more than 9000 synthetic social networks of different size, connectivity and network family, and run a simulator of pathogen spread on each one of those social graphs using a contact network model. We focus on analysing metrics of pathogen spread (e.g. total percentage of infected) for the generated networks, studying the effect of the network family, the degree and level of global connectivity, as well as many other topological network metrics. “Methods” section contains more information on the contact network model and the experimental setting for our simulations.

#### Contact network model

Our contact network method extends the standard compartmental SEIR model to stochastic networks. We consider a graph representing individuals (nodes) and their interactions (edges). At a given time, an individual makes contact with a subset of random individuals from their set of close contacts with certain probability and with a subset of individuals outside of their network with a different probability. That is, our model, which takes inspiration from previous work^12^, considers two different types of social interactions: (i) *Local interactions*, i.e. with close contacts – individuals with whom one has repeated and sustained interactions regularly, such as housemates, family members, close friends, etc. and (ii) *Global interactions*, i.e. with casual contacts – individuals with whom one has incidental or superficial contact infrequently (e.g., at the grocery store, at a public event, in the elevator). Global interactions are of the annealed kind^7^, changing dynamically in our simulation with each time step. These are represented in the models in the form of a parallel mode of mean-field global transmission. Local interactions are defined by the social graph, which is sampled from a network family, and are static throughout the simulation.

#### Network families

We consider four well-established classes of social networks: (i) random homogeneous networks, also known as Erdos Renyi, (ii) heavy tailed networks or scale-free, (iii) small world networks, as well as iv) community-based networks. These families of networks cover different aspects commonly observed in human interaction networks^13^, such as heterogeneity, heavy-tailed and broad degree distribution, transitivity, assortativity and community structure. See “Contact network SEIR modeling” section for a detailed description of these network families. The graph generator for scale-free and small-world networks has a hyper-parameter that increases and breaks clustering respectively. We experiment with it and generate both small-world and scale-free networks of different clustering. Additionally, with the community-based graph generator^14^ we cover a large range of the aforementioned aspects commonly observed in human interaction networks by doing a grid search of 6 hyper-parameters. More details on these hyper-parameters can be found in “Families of networks considered” section

#### Simulation settings

We have done a wide range of experiments with the mentioned families of networks for different network sizes (N=500, 1000 and 2000) and levels of connectivity (mean degree of 4, 10 and 20, with and without global interactions). The only network family for which we only consider N=1000 and degree of 10 is community-based networks, due to the wide range of the hyper-parameters that we experiment with for the graph generator (see “Families of networks considered” section). The main reason behind experimenting with relatively small network sizes was the algorithmic complexity of many of the network metrics considered, which grew rapidly with the number of nodes and edges in the network. However, by running a small subset of exploring simulations with larger network sizes (up to N=10000) we could not discriminate observable size effects. This led us to the design choice of focusing on smaller network sizes and using the available computing resources to calculate a larger set of network metrics (30 in this case) and conduct a thorough experimental design, where we sample social graphs from each configuration of the network family generator 30 times (thus computing the network metrics as well 30 times) and then running the pathogen spread simulator 10 times for each of these networks to account for stochasticity. This means that effectively for every network configuration (i.e. combination of network size and average degree, network family and specific configuration of the hyper-parameters of the graph generator) we run the simulator 300 times. However, the presence of observable size effects should be tested in further subsequent experiments, as it is possible that to properly study scale-free networks we may need to scale up to larger network sizes.

The simulator takes three pathogen parameters: probability of transmission $\beta$ given contact, rate of progression $\sigma$ and rate of recovery $\gamma$. These have been initialised to an estimation of the parameters for SARS-CoV-2 available when we started our simulations. Specifically, $\beta$ is set to 0.155, $\sigma$ to $\frac{1}{5.2}$ and $\gamma$ to $\frac{1}{12.39}$. Initial condition is set set it to 20 individuals (selected at random). Additionally, we would like to emphasize that we introduce the pathogen twice to avoid the situation of the infection extinguishing before reaching a critical size. We first run the simulation until the pathogen naturally dies off (or herd immunity has been achieved) and then reintroduce it by infecting 20 more susceptible individuals in the new social graph (which is now composed of susceptible and immunised individuals as opposed to the graph in the first simulation where all the individuals are susceptible). The total amount of infected individuals is the sum of infected for both simulations.

Importantly, for the sake of comparison, all simulations have factored exactly the same total of social interactions per time step, and this is independent of the level of local and global interactions, the network family and the average degree. More specifically, on average each individual makes one contact per time step (but this is only the average of the population, and will vary across individuals). We note that if increasing the number of social contact per individual per time step, the simulation simply progresses faster. In the case of local/global interactions, we always maintain the average number of interactions constant, and when increasing the probability of global interactions we decrease the probability of local interactions. Note that this is only a experimental choice that is maintained across all simulations so that we can compare the results across different families of networks and levels of global/local interactions easily, i.e. when the same number of social interactions occurr.

Our simulation considers a linear relationship between the average degree of an individual and the probability of social interaction. That is, the more distinct contacts one individual has, the larger the probability of interacting. This is because previous work has shown a super-linear association between the number of contacts and their duration^9^, indicating the possibility that super-spreaders need to be defined not only in number of connections but also in intensity.

#### Network metrics

For each synthetic social graph that we generate, we compute 30 different topological network metrics (i.e. that only use the adjacency matrix), which we categorise in global and local metrics depending on whether they are computed only on the basis of nodes’ neighbours (local), or using the whole graph (global). These can be categorised under:

- *Distance metrics*: Wiener index, diameter, radius, local efficiency, global efficiency, eccentricity, closeness and betweenness. From these last 3 we compute the average, std and skewness.
- *Connection metrics*: degree metrics (avg. degree, std. degree, max degree, min degree, branching factor, skewness degree, kurtosis degree, entropy degree), is connected, number of connected components, assortativity, clustering, transitivity and node connectivity.
- *Spectral metrics*: algebraic connectivity and spectral radius.

Local metrics are all degree metrics, assortativity, clustering, local efficiency and transitivity. Global metrics are the remaining ones.

Our work finds that the three most predictive metrics of the spread are global efficiency, average closeness and algebraic connectivity. We describe now these network metrics:

- *Global efficiency*: The efficiency of a network aims to capture how efficiently it exchanges information^15^, where the intuition is that the more distant two nodes are, the less efficient communication will be. In particular, global efficiency quantifies the exchange of information across the whole network by averaging pairwise efficiencies between nodes. Its formal definition assumes that efficiency is inversely proportional to the distance between pairs of nodes.
- *Algebraic connectivity*: The algebraic connectivity of a graph is the second smallest eigenvalue of its Laplacian matrix. This eigenvalue is greater than 0 only for connected graphs. The magnitude of this metric reflects how well connected the overall graph is. It is considered to be a lower bound on node-connectivity. An increase in algebraic connectivity seems to correlate well with a decrease in the characteristic path length of networks - which would result in quicker communication through the network^16^. This metric plays a crucial role in dynamic phenomena such as diffusion processes, synchronization stability, and network robustness.

### Not all social interactions have equal impact on transmission

**Figure 1:**
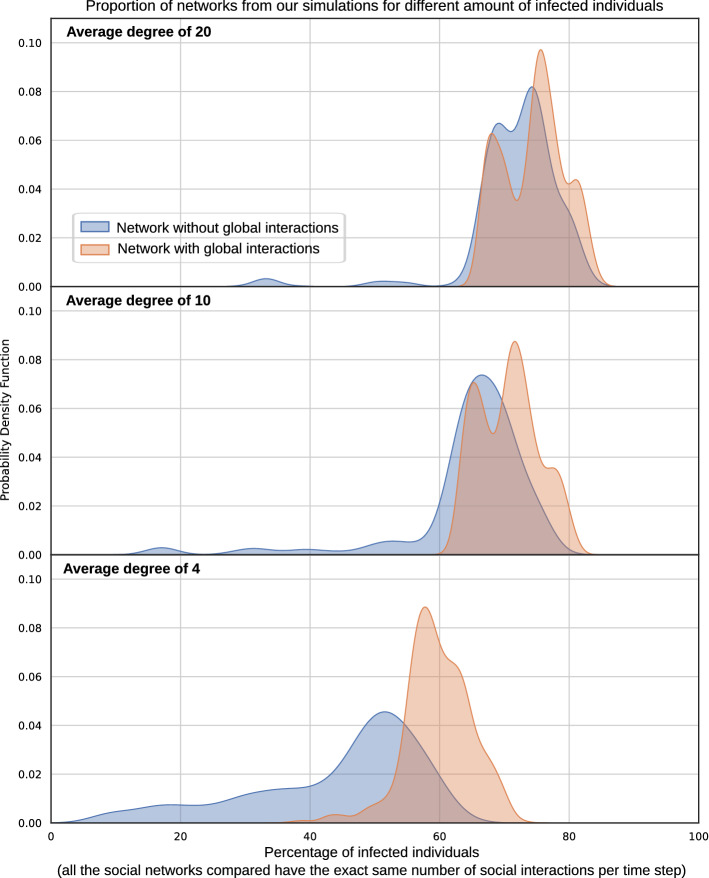
The impact on spread of global interactions and different average degrees, aggregating across networks of all sizes and diverse network topologies. In networks without global interactions, individuals interact exclusively within their familiar social group. In networks with global interactions, individuals interact with strangers with a probability of 0.2 at each time step, the remainder (0.8) confined to their social group. Importantly, for all the compared networks in this plot (independently of globality and average degree), there is exactly the same number of social interactions per time step. This plot aggregates networks of all sizes (500, 1000 and 2000) and features all Erdos-Renyi, small world and scale-free networks generated. For average degrees of 20 and 10 the probability density function shows differentiated peaks. The reason for this is that we do not consider all possible network sizes and average degrees in our simulations, but instead aggregate all results over our generated networks with 500, 1000 and 2000 nodes and degrees of 4, 10 and 20. Thus, the peaks are associated to different configurations. We believe that if we generated networks across the whole space of possibilities, these peaks would not occur. In fact, that is the case when we show the results for one specific configuration, as the results in Fig. 3 show.

Our extensive simulations ($>100\,000$ runs) show that diverse families of networks with the same average degree can vary widely in their disease burden (Fig. 1), in agreement with previous results in the literature^12^. Specifically, we show that even for a fixed average degree the spread can change drastically, i.e. from 10% to more than 60% infected for average degree of 4, and from 30% to more than 80% for average degree of 20. As can be seen in Fig. 1, this difference is particularly significant when the average degree is low (e.g. degree of 4).

The second major insight is that not all human interactions have equal impact on spread^10,11^. Namely, there is an important difference between i) networks of low vs. high average degree and ii) networks with/without global interactions. Spread increases for all networks that are globally connected and have high average degree, even when the volume of social interactions per time step is kept constant for all simulations. Again, global interactions have the greatest impact on networks of low degree. This striking variation reflects a neglected truth – social contacts differ in their effect on spread.

Our results also validate previous findings in the literature that networks with the same volume of interaction per time step yield different levels of disease burden dependent on the form, not just the volume of observed connectivity. What we call in this paper local interactions (included in our simulations as sustained contact with one’s close social network), seem to have in general lower impact on overall spread^11^.

### Certain network geometries are resilient to pathogen spread

**Figure 2:**
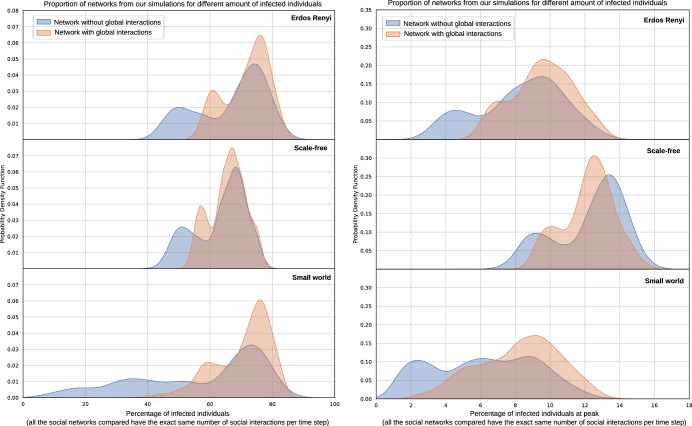
The impact on spread (total percentage - left, peak of infection - right) of different network families. In this plot we only consider unadulterated scale-free and small world networks (i.e. without increasing or breaking its clustering in the graph generator, since this significantly changes their original properties, often bringing them closer in terms of results to Erdos Renyi. The plot aggregates networks of all sizes and average degrees. The peaks in the probability density function are associated to different configurations.

We investigate now the effect of network families on the infection spread (Fig. 2). We observe that Erdos Renyi networks often overestimate infections when compared to networks that have been seen to share features with some real-world social networks (e.g. in terms of degree heterogeneity for scale-free or clustering for small world). Scale-free often leads to less infections than Erdos Renyi but a higher peak of infection. This may be not intuitive at first, since scale-free networks represent networks with nodes with very high connectivity (e.g. super-spreaders). However, these nodes have been shown to get infected quickly and then become immune, and given that they are highly connected they help slow down the spread^7^. This obviously assumes total immunization after infection. It is noteworthy in any case that the infection progresses more quickly and a higher peak is achieved for scale-free networks, even when the total number of infected is lower. Small world networks are the most resilient to infections, especially for simulations without global interactions. As it is expected, adding random global interactions to both scale-free and small world networks make these closer to Erdos-Renyi networks, and this is reflected in our results. This reinforces that, as shown previously in the literature, at fixed average degree some network topologies are less vulnerable to transmissible diseases than others. Specifically, there exist network geometries which show a natural resilience to the spread. These cases are worth studying in order to devise real-world interventions, potentially shedding light on how to reduce the spread of the pathogen more effectively.

**Figure 3:**
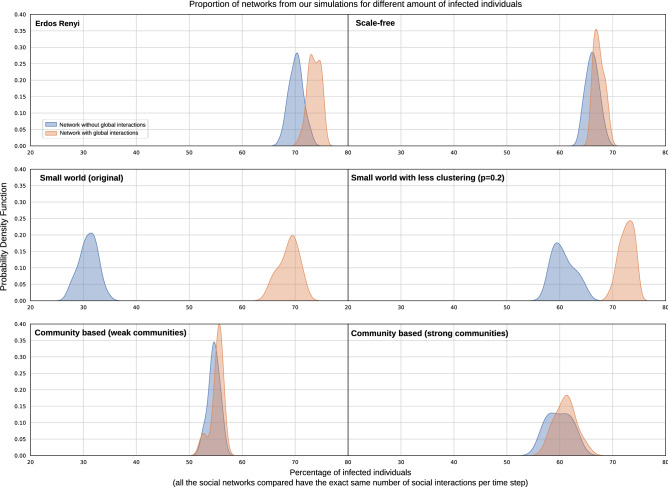
The impact on spread of all network families (including community-based) for networks with 1000 nodes and an average degree of 10. In this plot we only consider unadulterated scale-free and small world networks (i.e. without increasing or breaking its clustering in the graph generator, since this significantly changes their original properties, often bringing them closer in terms of results to Erdos Renyi). We only plot two hyper-parameter configurations for community-based networks, since results differ widely based on the hyper-parameter configuration. We choose community-based networks with 50 communities and set transitivity and degree correlation to the default values of the graph generator (hyper-parameter $a=0.5$ and $g=0.8$ respectively). The community-based networks in the left part of the plot represent weaker communities, where the probability of edges to be formed within communities (hyper-parameter *b*) is 0.3, a node can belong to 25 different communities (hyper-parameter *r*) and the probability of a node belonging to the multiple communities is 0.5 or 0.7 (hyper-parameter *q*). The community-based networks in the right part of the plot represent stronger communities, where the probability of edges to be formed within communities (hyper-parameter *b*) is 0.8, a node can only belong to 5 different communities (hyper-parameter *r*) and the probability of a node belonging to the multiple communities is 0.1 or 0.3 (hyper-parameter *q*).

Fig. 3 shows the comparison to a selection of community-based networks, where we only consider networks with N=1000 and an average degree of 10. We can see again that Erdos Renyi shows a higher percentage of infected than the rest of network families. Small world are the most affected by adding global interactions. Community-based networks show in general lower infections than Erdos Renyi, scale-free and certain small-world networks. However, each of the hyper-parameters that we experiment with in community-based networks have a different effect on the spread. For example, we observe from our results that a negative degree correlation in community-based networks leads to higher percentage of infections, as highly connected nodes connect to less connected individuals, reaching even those that may have a lower degree and be more isolated. Additionally, community-based networks seem more resilient to global interactions. The reason behind this is worth exploring in future work. We hypothesize that these networks may still have weakly connected components, and adding 20% of global connections may not change that drastically (especially if communities are large and many of those 20% of interactions could still be within communities, as opposed to across communities). Additionally, stronger connected communities generally show higher percentages of infection than weaker connected community-based networks. We hypothesize that because of the strong connectedness of the different communities the infection reaches most nodes within the community, and since we introduce effectively 40 infected individuals at random in the social graph, this could cover most of the communities (in the case of Fig. 3 we show the results for 50 communities). We continue this discussion on community-based networks in the next section, where we show that these network metrics show generally low algebraic connectivity, which is one of the metrics that we found to be most predictive of the spread.

### A combination of network metrics can accurately predict spread

**Figure 4:**
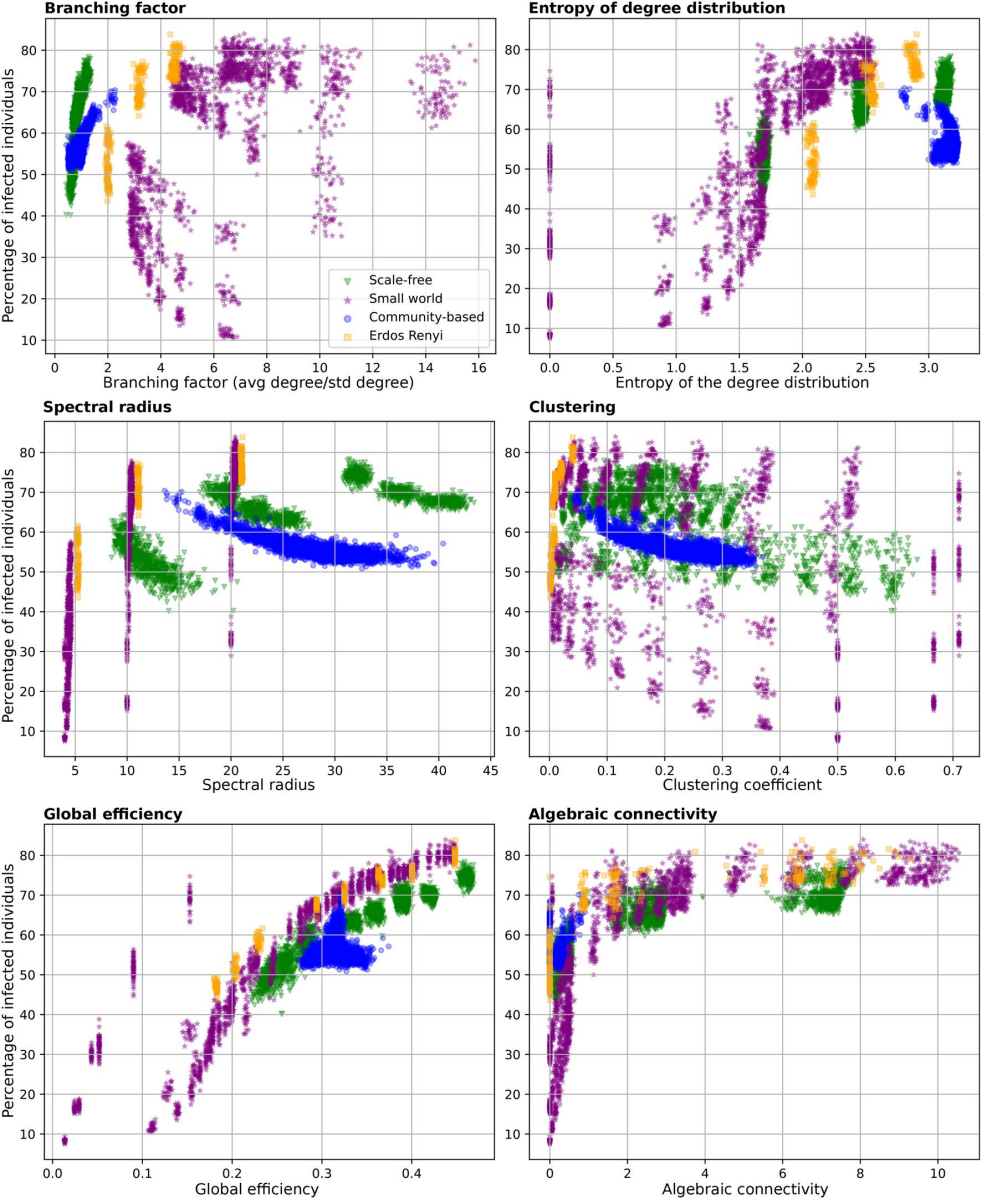
Scatter plots of a selection network metrics vs the cumulative percentage of infected. Each point represents a social network. This plot includes all the networks generated in this study. Colours and markers depict different network families. Note that for community-based networks we only run the case of N=1000 and average degree of 10.

Fig. 4 shows a scatter plot of the relationship between several network metrics and the cumulative percentage of infected, using all of our generated networks. Different network families have been highlighted in different colours and markers. The scatter plot clearly shows that topology metrics previously overlooked in the literature, such as global efficiency and algebraic connectivity show a stronger relationship than other metrics traditionally proposed as determinant in the literature and considered in policy making, such as metrics of the degree distribution or the spectral radius (Fig. 4). It is also noteworthy that some of the found relationships in the literature may hold for specific network families. For example, see the positive relationship between branching factor and percentage of infected for scale-free networks (highlighted in green). This is not the case, however, for small-world networks. This is one of the main conclusions of our study and the one we would like to highlight the most. While our analysis points to global efficiency and algebraic connectivity as the most predictive metrics of the spread considering 4 families of network topologies, further experiments with additional social graphs may highlight other network metrics as predictive. In this sense, we show the importance of considering all network metrics together using multiple social network configurations, rather than simply varying the metric of interest alone in controlled numerical experiments or analytical calculations.

**Figure 5:**
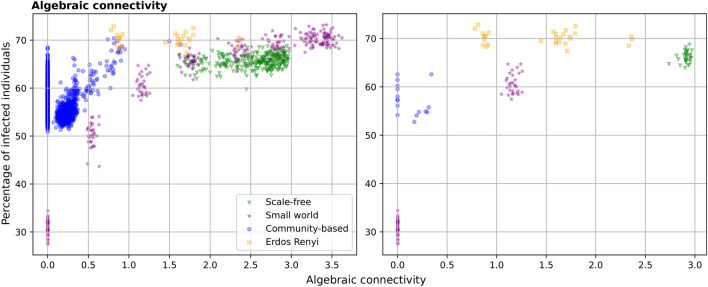
Scatter plots of algebraic connectivity vs the cumulative percentage of infected for a selection of networks. This plot only includes networks with N=1000 and degree of 10. The left part of the plot shows all potential network configurations, whereas the right part shows exactly the networks considered in Fig. 3.

Fig. 4 also shows that there exist hubs of points associated to different network families, following a similar trend in the network metric of choice and the percentage of infected. We have validated that these correspond to different configurations (e.g. N=500 vs N=1000, or different hyper-parameter configurations). The case of global efficiency is of particular interest, where we can appreciate certain groups of small world networks with much lower global efficiency. These correspond to unadulterated/original small world networks, i.e. for which we do not change the clustering coefficient in the graph generator. Finally, it is also noteworthy that community-based networks show in general a low algebraic connectivity, which is one of the most predictive metrics of the spread. To connect this to the results in the previous “Certain network geometries are resilient to pathogen spread” section) we now plot algebraic connectivity for a selection of networks (N=1000 and degree of 10) in Fig. 5. The left part of the plot shows all potential configurations, whereas the right part shows exactly the networks displayed in Fig. 3. The low algebraic connectivity of community-based networks could explain the lower infection rates shown previously. There are some networks that do not follow this trend and for which algebraic connectivity is zero. This is the case of networks with disconnected components, which seems to be the case in the community-based networks from Fig. 3 with strong communities. Still, the relationship between algebraic connectivity and infected is far from perfect. However, as we will show next, the variability of infected can be captured with a combination of network metrics.

**Figure 6:**
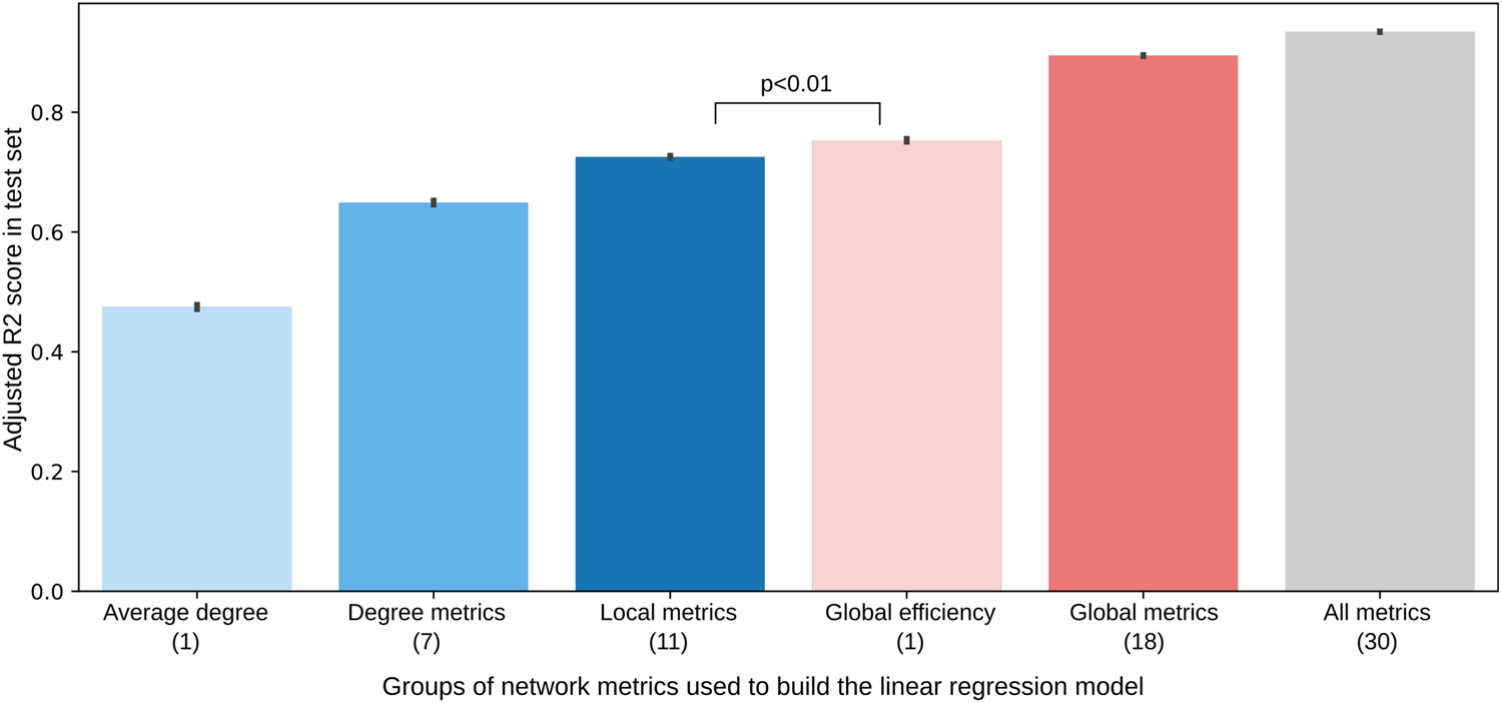
Bar chart of the test set performance (y-axis, measured as adjusted R2 score) of the regression models built on groups of features (x-axis, each group including in parenthesis the total number of features). The chart includes the confidence intervals computed over 100 random data holdouts. All comparisons are statistically significant for p<0.01.

To understand the impact of different network metrics on pathogen spread we performed a linear regression analysis (Fig. 6), building predictive models of pathogen spread based on subsets of topological network metrics. This is, the dependent variable in our analysis is the average percentage of infected individuals per network configuration. The independent variables are different subset of network metrics. We report adjusted $R^2$ computed on the test set of 100 random data holdouts with 80% of data for training and 20% for testing. When considering all 30 metrics, the linear regression model obtains an adjusted $R^2$ of 0.934 (in comparison to a nonlinear predictor which obtains 0.987). The small confidence intervals suggest highly stable results. The first insight is thus that a combination of the selected network metrics can accurately predict the variability of the spread. The remaining variance of the data could be partly due to the stochasticity of the simulation. Importantly, this figure also shows the predictive power of different groups of metrics. Specifically, this figure first shows that average degree alone may not be enough to predict the spread accurately. In fact, considering additional degree metrics (skewness, entropy, etc.) improves the predictive performance ($R^2$ from 0.473 to 0.631), further improved when taking into account the remaining local metrics ($R^2$ from 0.631 to 0.710). However, the model built using only global efficiency achieves better performance than the model that uses all degree metrics and the model that uses all local metrics ($R^2$ of 0.747). Additional global metrics complement global efficiency and increase the performance further. The data has strong multicollinearity, precluding us from drawing further conclusions from the model coefficients. All of these metrics that seem to have the highest influence on the spread relate to the path lengths between nodes and global connectivity of the social graph. Both contact tracing or mobility data that are already being recorded can be used for example to infer path lengths in different societies^17–19^ and thus restrict the social graph of simulations used in policy making. The findings in our work should be tested with larger size networks, which may represent real-world patterns better.

## Discussion

Our simulations highlight the importance of modeling a wide range of parameters of a social network, which can be used as a representation of its topology. Specifically, our work has shown across a full range of empirically-informed plausible network configurations that spread outcomes can be reliably predicted from a regression model built using the topological properties of the underlying social network. Our experiments with synthetic social networks have also validated that network metrics that were found to be predictive of the spread in the literature, are not so for all of our network configurations. Instead, our results point to other novel metrics. Among other research findings, our simulations also show (i) how much the topology of social networks can highly influence pathogen spread, (ii) that reducing casual random social contact reduces significantly the spread, (iii) that small world network topologies are resilient to infections, (iv) the usefulness of a principled multivariate approach for evaluating metrics predictive of the spread and (v) the superior predictive performance (with regard to the spread) of metrics that relate to the graph path length over metrics of the degree distribution. Our results also indicate that the effect of a social intervention could depend on the underlying social network. We observe this when reducing the average degree for a wide variety of network families. This is of crucial importance. A social intervention will almost always vary in its effect on each property of a social network. For example, closing public spaces reduces long range connectivity but not necessarily short range connectivity. Social interventions can be then seen as transitions from one complex social network organisation to another, not merely attenuations or amplifications of any given network. As different previous works have already pointed out, to maximise the impact of social interventions, we need complex, prescriptive models of social networks.

Our results point to the question of what are the network metrics that represent the real world and whether our conclusions extend to those networks. Although some studies and datasets aimed at this exist^20^, these works often focus exclusively on reporting metrics such as the average of the degree distribution or its moments. Instead, we have shown that we need reliable estimators of other crucial network metrics to characterise the spread. The space of optimal interventions could be large, diverse, and non-intuitive: the same intervention can have radically different effects across different social networks, and the same outcome may require radically different interventions. Given the observed wide space of scenarios, interventional inference is likely to require an exploration of the graph properties of the networks scenarios assessed to derive maximal efficient change in those properties that leads to the desired optimal outcome. It is thus clear that we need a wide range of complex network analysis epidemiological tools, as well as a platform that allows to do so systematically. Even if we do not know what are the topologies of real-world networks (and this certainly can change across societies and regions), it is worth exploring the space of complex interactions scenarios in a systematic manner. This could lead to a subset of interventions that perhaps collapse in trend regardless of the network and help restrict the decision making space.

We do not claim to have found the single network topological determinants of pathogen spread that apply to every situation. Instead, we aimed to show the importance of considering all network metrics simultaneously, as well as multiple families of social networks, when the objective is to find metrics that are predictive of the spread. In practical terms, our integrative framework could be easily extended to consider additional social graph generators, contact network models and network metrics. Future work should also test whether our findings apply to a wide range of pathogen parameters since epidemic models are non-linear. In the real world, determining a complete mixing network requires knowledge of every individual in a population and every relationship between individuals. For all but the smallest populations, this is an impractically time-consuming task. However, topological determinants can be captured from a subgraph of the population instead of the whole network, and as we have shown, these could be crucial for estimating infections. They may be useful to design more accurate simulations and to recommend interventions or to target/correct aspects of connectivity that will actually control infection spread, while maintaining economic activity. We believe that further study into the effect of different social interventions on the spread and global efficiency of the network are needed. We think that such a line of research could potentially lead to crafting measures that define the optimal interventions in terms of spread per social contact.

## Methods

### Compartmental modelling

Compartmental models subdivide host populations by disease status. The adjective compartmental comes from viewing the disease states as compartments into and out of which individuals move throughout the epidemic. This is the case of a simple and widely used model named SEIR, which tracks the movement of hosts among four states: (1) susceptible (S), meaning that the individual has never had the disease and is susceptible to being infected; (2) exposed (E), meaning that the individual has been infected but is not yet infectious; (3) infected (I), meaning that the individual currently has the disease and can infect other people; and (4) resistant (R), meaning that the individual does not have the disease, cannot infect others, and cannot be infected.

The model then evolves in discrete time steps^21^: (1) Each susceptible individual draws a uniformly random person from the population. If the person drawn is infected, then the susceptible individual changes their state to infected with probability $\beta$. However, for many important infections, there is a significant latency period during which individuals have been infected but are not yet infectious themselves. During this period the individual is in compartment E (for exposed). This latency period is represented by parameter $\sigma$. (2) Each infected individual changes their state to resistant with probability $\nu$. (3) Each resistant individual remains resistant. This model makes several important assumptions, e.g. infected hosts are assumed to have contacts with random individuals from the population according to a Poisson process that yields an average contact rate of $\beta$ per unit time. Disease transmission then occurs if and only if the individual at the receiving end of the contact is susceptible. Infectious hosts leave the infectious state at an average rate $\nu$ either by recovering and becoming immune or by dying. In the limit of a large host population, this process can be modeled by the following coupled nonlinear differential equations:

1 $$\begin{aligned} \frac{dS}{dt} = - \beta SI, \;\;\; \frac{dE}{dt}= \beta SI-\sigma E, \;\;\; \frac{dI}{dt} = \sigma E - \nu I, \;\;\; \frac{dR}{dt} = \nu I, \end{aligned}$$

where *S*(*t*), *I*(*t*), *E*(*t*) and *R*(*t*) are the numbers of susceptible, infected, exposed and recovered hosts, respectively. The model ignores the birth and death of susceptibles, the total population size *N* is static.

Although compartmental SEIR models have proven to be quite useful in modeling epidemics, they often do not properly model some important aspects of disease spread, since they assume a fully mixed, homogeneous population which may not adequately reflect reality.

### Contact network SEIR modeling

We apply the standard compartmental SEIR model to stochastic networks. To do so, we consider a graph *G* representing individuals (nodes) and their interactions (edges). Each node individual *i* in the graph has associated a current state: state $X_i$ can be *S* (susceptible), *E* (exposed), *I* (infected) or *R* (recovered). At a given time, an individual *i* makes contact with a subset of random individuals from their set of close contacts (denominated local interaction) with probability $p_l$ and with a subset of individuals outside of their network (called global interactions) with probability $p_{g}$. More specifically, our model (inspired by^12^), considers two different types of contacts:

- *Close contacts* – individuals with whom one has non-cursory (e.g., repeated, sustained, and/or physical) interactions on a regular basis, such as housemates, family members, close coworkers, close friends, etc. This set of close contacts the population is defined through the contact network.
- *Casual contacts* – individuals with whom one has incidental, brief, or superficial contact on an infrequent basis (e.g., at the grocery store, on transit, at a public event, in the elevator) – are also represented in these models in the form of a parallel mode of mean-field global transmission.

Previous work has found an average of 13.4 contacts per day per person consistently for different European countries^20^. However, people will make changes in behavior (e.g. reduce their number of contacts) in response to knowledge of an epidemic. These changes will not only reduce the number of contacts of the entire population, but also change the mixing patterns in the population, which is why we experiment with different average degree and types of human interactions.

When a susceptible individual interacts with an infectious individual they become exposed with probability $\beta$ and transition towards infected with rate of progression $\sigma$. The model takes three pathogen parameters: probability of transmission $\beta$ given contact, rate of progression $\sigma$ and rate of recovery $\gamma$. These have been initialised to estimated parameters for SARS-CoV-2.

The probability of transitioning from exposed to infected and from infected to recovered remain the same than in the standard SEIR model (described in more detail in “Results” section). However, the probability that a susceptible individual *i* moves to the exposed state needs to be specified by the contact network of the individual. We define it as:

2 $$\begin{aligned} \texttt {Pr}(X_i = S \rightarrow E) = \left[ \beta \cdot B_i \left( p_g\cdot \frac{ \sum _{z=1}^N \delta (X_z = I) }{N} + p_l \cdot \frac{ \sum _{j \in C_i} \delta (X_j = I) }{|C_i|} \right) \right] \delta (X_i = S), \end{aligned}$$

where $\delta$ is an indicator function such that $\delta (X_i=A)=1$ if the state of $X_i$ is A, or 0 if not, $C_{i}$ denotes the set of close contacts of node i and $B_i$ is a factor accounting for the budget of interactions of individual *i* to its contact network at each time step. The terms $\frac{ \sum _{z=1}^N \delta (X_z = I) }{N}$ and $\frac{ \sum _{j \in C_i} \delta (X_j = I) }{|C_i|}$ thus correspond respectively to the percentage of infected individuals in the population and in the contact network of individual *i*, respectively. The product of the network locality parameters ($p_l$ and $p_g$) and the transmission parameter set the weight of transmission among close (local) and casual (global) contacts in the modeled population.

If $B_i$ is set to 1, then individual *i* makes contact with exactly 1 individual each time step of the simulation (either in the contact network or outside of it with probabilities $p_l$ and $p_g$). However, this makes the assumption that individuals highly connected have the same interactions per time step than individuals with a small contact network. In other words, it assumes an equal budget of interactions per individual, which may not be realistic in the case of super-spreaders. In fact, previous work has shown a super-linear association between the number of contacts and their duration^9^, indicating the possibility that super-spreaders need to be defined not only in number of connections but also in intensity. This is, the more distinct interactions one individual has, the larger is the average time dedicated to those interactions.

To appropriately compare the spread, we maintain the total number of interactions across the population per time step. We set $B_i = \frac{|C_i|}{{\bar{k}}}$, where ${\bar{k}}$ is the average degree for the population. This sets a linear relationship between $B_i$ and $|C_i|$, with individuals with larger contact networks having a larger interaction budget, while still maintaining the total number of interactions for the whole population. We additionally experiment with different $p_l$ and $p_g$, to see the difference that local/global interactions have on the spread.

#### Characteristics of human interaction networks

There are some properties that are believed to be representative of human interaction networks. Note that not all networks need to fulfill all, and this may vary across different societies:

- *Heterogeneity*: The degree (size of the contact network per individual) varies across individuals and groups of like-individuals (e.g., age groups). Groups of individuals may differ in the numbers of within- and between-group contacts they make.
- *Broad degree distribution*: Most individuals have roughly average connectivity (degree), but there is individual variation around the mean degree (this is in contrast with, scale-free networks where most individuals have very low degree and the mode is often well below the mean).
- *Heavy-tailed degree distribution*: A small number of individuals have many more contacts than average, so the degree distribution tends to have a relatively long right tail.
- *Assortativity*: There tends to be correlation in degree between adjacent nodes in the contact network. That is, highly-connected individuals tend to have highly-connected contacts.
- *Transitivity (or clustering)*: Individuals A and B are relatively likely to be contacts of each other if they both share a mutual contact C.
- *Community Structure*: Contact networks often have communities of individuals (groups of nodes) that are more likely to be contacts of each other than they are to be with individuals from another community.

#### Families of networks considered

We consider four classes of social networks: (i) *random homogeneous networks*, also known as *Erdos-Renyi*, (ii) *heavy tailed networks or scale-free*, (iii) *small world networks* and (iv) community-based networks. Part of these networks have been shown to be the representative of a variety of animal and human social networks^13^ and cover some of the characteristics outlined:

- *Random homogeneous networks* are the underlying assumption of most compartmental models. The degree distribution can be approximated by a Poisson and peaks around the average, thus denoting a statistical homogeneity of the nodes. These networks are governed only by stochasticity and thus do not represent any structural properties that one would expect from a real world network, such as a high clustering coefficient^12^. To create Erdos Renyi networks, we choose a graph uniformly at random from the set of all graphs with N nodes and e edges.
- *Scale-free networks* are characterized by a highly skewed distribution of contacts such that most of the nodes are weakly connected and a small number have very high connectivity^22^. There is empirical evidence that in fact some real-world networks exhibit such a skewed degree distribution^7,23^, varying over several orders of magnitude, although this is still debated for contact networks^1,24^. The degree distribution of these heterogeneous networks can often approximated by a power-law behavior, which implies a non-negligible probability of finding nodes with very large degree. We use Holme and Kim algorithm^25^ for growing graphs with powerlaw degree distribution and approximate average clustering. It is essentially the Barabási-Albert (BA) growth model with an extra step that each random edge is followed by a chance of making an edge to one of its neighbors too (and thus a triangle). This probability of adding a triangle after adding a random edge is set as a hyper-parameter (*p*). We experiment with *p* from 0.0 to 0.8 (in steps of 0.1).
- *Small world networks* are characterized by a degree distribution that is roughly symmetric about the mean, with a high degree of node clustering and a short characteristic path length^26^. This model, although better suited for social networks with high clustering coefficient, has a degree distribution and centrality measures decaying exponentially fast away from the average value. The small-world model thus generates homogeneous networks where the average of each metric is a typical value shared by all nodes of the network and with little variation. We use Watts-Strogatz small-world model, generating a connected graph by repeated generation of graphs. This graph generator has a hyper-parameter (*p*) that sets the probability of rewiring each edge at random. We experiment with *p* from 0.0 to 0.8 (in steps of 0.1).
- *Community-based networks*: These other well-known families of networks (random homogeneous, scale-free and small world) do not cover all the aspects outlined for human interaction networks. To generate a more diverse set of networks we additionally use a community-based graph generator^14^ that allows us to generate networks with all these parameters. Specifically, we perform a grid search of different values for the following 6 hyper-parameters: (i) Number of communities (*k*), (ii) strength of those communities (probability of edges to be formed within communities (*b*), (iii) number of communities a node can belong to (*r*), (iv) the probability of a node belonging to the multiple communities (*q*), (v) the strength of degree similarity effect on edge formation (*g*) and (vi) the strength of common neighbor’s effect on edge formation edges (*a*). We experiment with *k* in [1, 5, 10, 50, 100], *b* in [0.3, 0.5, 0.8], *r* in [0.1, 0.25, 0.33, 0.5, 1] as a percentage of the total number of communities, *q* in [0.1, 0.3, 0.5, 0.7], *g* in $[-0.3, -0.1, 0.2, 0.5, 0.8]$ and *a* in [0.0, 0.2, 0.5, 0.8].

#### Network metrics computed

We have computed 30 different topological network metrics (i.e. that only use the adjacency matrix) for each of the network graphs. We categorise these metrics in global and local metrics depending on whether they are computed only on the basis of nodes’ neighbours (local), or using the whole graph (global). For more information on how each metric is computed please refer to the code associated with this project.

## Data Availability

The code and metrics for all network topologies are available at this project’s github page https://github.com/mperezortiz/topology_spread.

## References

[1] Bansal S, Grenfell BT, Meyers LA (2007). When individual behaviour matters: homogeneous and network models in epidemiology. J. R. Soc. Interface.

[2] Block, P. *et al.* Social network-based distancing strategies to flatten the covid-19 curve in a post-lockdown world. *Nat. Human Behav.* **4**, 588–596 (2020). 10.1038/s41562-020-0898-6 32499576

[3] Meyers L (2007). Contact network epidemiology: bond percolation applied to infectious disease prediction and control. Bull. Am. Math. Soc..

[4] Volz EM, Miller JC, Galvani A, Meyers LA (2011). Effects of heterogeneous and clustered contact patterns on infectious disease dynamics. PLoS Comput. Biol..

[5] Miller JC (2009). Spread of infectious disease through clustered populations. J. R. Soc. Interface.

[6] Anderson RM, May RM (1992). Infectious diseases of humans: dynamics and control.

[7] Pastor-Satorras R, Vespignani A (2001). Epidemic spreading in scale-free networks. Phys. Rev. Lett..

[8] Hethcote HW, Yorke JA (2014). Gonorrhea transmission dynamics and control.

[9] Cattuto C (2010). Dynamics of person-to-person interactions from distributed rfid sensor networks. PLOS ONE.

[10] Zhang J (2020). Changes in contact patterns shape the dynamics of the covid-19 outbreak in china. Science.

[11] Brethouwer, J.-T., van de Rijt, A., Lindelauf, R. & Fokkink, R.“Stay nearby or get checked”: A covid-19 control strategy. *Infect. Dis. Model.* **6**, 36–45 (2021). 10.1016/j.idm.2020.10.013. 10.1016/j.idm.2020.10.013 PMC7669247 33225114

[12] Pastor-Satorras R, Castellano C, Van Mieghem P, Vespignani A (2015). Epidemic processes in complex networks. Rev. Modern Phys..

[13] Sjöberg M, Albrectsen B, Hjältén J (2000). Truncated power laws: a tool for understanding aggregation patterns in animals?. Ecol. Lett..

[14] Fagnan, J., Abnar, A., Rabbany, R. & Zaïane, O. R. Modular networks for validating community detection algorithms. *CoRR***abs/1801.01229** (2018). arXiv:1801.01229.

[15] Latora V, Marchiori M (2001). Efficient behavior of small-world networks. Phys. Rev. Lett..

[16] Jamakovic, A. & Uhlig, S. On the relationship between the algebraic connectivity and graph’s robustness to node and link failures. In *2007 Next Generation Internet Networks*, 96–102 (IEEE, 2007).

[17] Mistry D (2021). Inferring high-resolution human mixing patterns for disease modeling. Nat. Commun..

[18] Klein, B. *et al.* Reshaping a nation: Mobility, commuting, and contact patterns during the covid-19 outbreak. *Northeastern University-Network Science Institute Report* (2020).

[19] Kraemer MU (2020). The effect of human mobility and control measures on the covid-19 epidemic in china. Science.

[20] Mossong J (2008). Social contacts and mixing patterns relevant to the spread of infectious diseases. PLoS Med..

[21] Dimitrov, N. B. & Meyers, L. A. Mathematical approaches to infectious disease prediction and control. In *Risk and optimization in an uncertain world*, 1–25 (INFORMS, 2010).

[22] Barabási A-L, Albert R (1999). Emergence of scaling in random networks. Science.

[23] Liljeros F, Edling CR, Amaral LAN, Stanley HE, Åberg Y (2001). The web of human sexual contacts. Nature.

[24] Broido AD, Clauset A (2019). Scale-free networks are rare. Nat. Commun..

[25] Holme P, Kim BJ (2002). Growing scale-free networks with tunable clustering. Phys. Rev. E.

[26] Watts DJ, Strogatz SH (1998). Collective dynamics of ‘small-world’ networks. Nature.

